# Spontaneous emergence of counterclockwise vortex motion in assemblies of pedestrians roaming within an enclosure

**DOI:** 10.1038/s41598-022-06493-0

**Published:** 2022-02-16

**Authors:** Iñaki Echeverría-Huarte, Alexandre Nicolas, Raúl Cruz Hidalgo, Angel Garcimartín, Iker Zuriguel

**Affiliations:** 1 Departamento de Física y Matemática Aplicada, Facultad de Ciencias, Universidad de Navarra, 31080 Pamplona, Spain; 2 Institut Lumière Matière, CNRS & Université Claude Bernard Lyon 1 & Université de Lyon, 69622 Villeurbanne, France

**Keywords:** Applied physics, Statistical physics, thermodynamics and nonlinear dynamics

## Abstract

The emergence of coherent vortices has been observed in a wide variety of many-body systems such as animal flocks, bacteria, colloids, vibrated granular materials or human crowds. Here, we experimentally demonstrate that pedestrians roaming within an enclosure also form vortex-like patterns which, intriguingly, only rotate counterclockwise. By implementing simple numerical simulations, we evidence that the development of swirls in many-particle systems can be described as a phase transition in which both the density of agents and their dissipative interactions with the boundaries play a determinant role. Also, for the specific case of pedestrians, we show that the preference of right-handed people (the majority in our experiments) to turn leftwards when facing a wall is the symmetry breaking mechanism needed to trigger the global counterclockwise rotation observed.

## Introduction

From raging hurricanes sweeping through countries down to microscopic spirals formed by confined bacterial colonies, self-organised vortices are found at very different scales in Nature. It is well known that hurricanes and tornadoes rotate counterclockwise (CCW) or clockwise (CW) depending on the Hemisphere, owing to the Coriolis force. While vortical structures have also been observed in a variety of other systems at much smaller scales^1^, and in particular in active assemblies^2–6^, little attention has been paid so far to the direction of rotation. Indeed, the formation of vortex structures is an intriguing feature per se. Admittedly, manifestations of collective motion are widespread in systems composed of many active bodies, whether it be milling patterns in fish schools^7^, the stable coherent vortices in their confined assemblies of active rollers^4^, or the circle pits formed by heavy-metal concert attendees^6^. In all these cases, a flocking-like term^8^ promoting alignment among neighbours was central in the rationalisation of the observations. On the contrary, in their endeavours to reproduce the spirals formed in dense bacterial suspensions, Lushi et al.^9^ argue that the steric repulsions between the bacteria, together with the confined settings, are enough to induce some (limited) degree of spiralling circulation.

To our knowledge, none of these *models* led to a preferred direction of rotation, breaking the CW-CCW symmetry, even though it was empirically observed in some cases; for instance, the vast majority (95%) of real circle pits in^6^ do rotate in the CCW direction. Here, we experimentally reproduce this behaviour in sparse pedestrian assemblies and develop a minimal model that is able to reproduce the most salient features of the collective motion obtained.

## Results

### Counterclockwise vortex motion in pedestrian experiments

**Figure 1:**
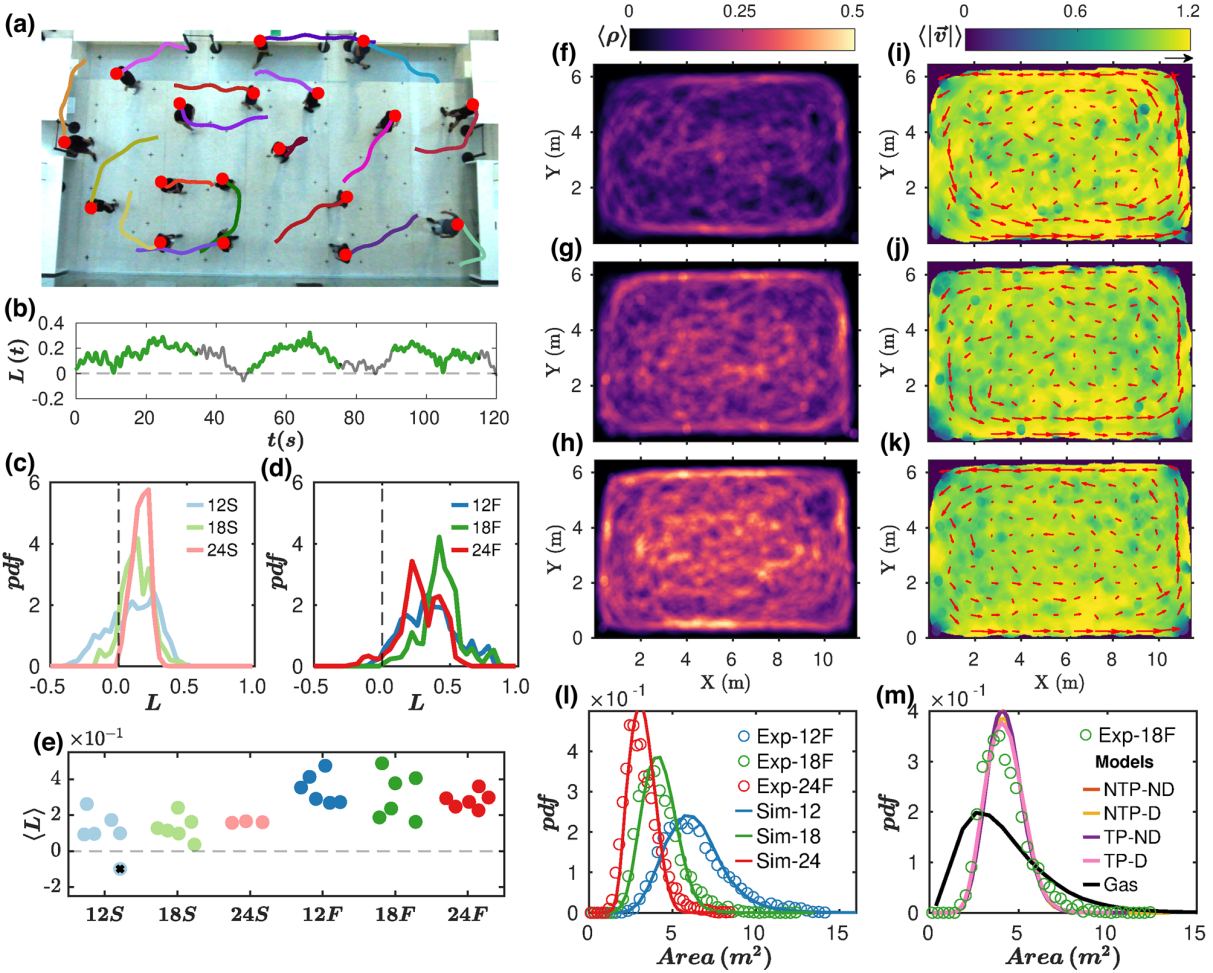
Counterclockwise vortex motion in pedestrian experiments. (**a**) Snapshot of 18 pedestrians moving “fast” within the arena. The tails show 2-s trajectories. (**b**) Temporal evolution of the instantaneous angular momentum $L(t)$ averaged over all pedestrians for the experiment shown in A. The time intervals when the movement is “free” (the ones analyzed in this work) are shown in green. (**c**,**d**) Distributions of the instantaneous angular momentum $L(t)$ for 12, 18 and 24 pedestrians walking slow (S) in C, and fast (F) in D. (**e**) Temporal average of $L(t)$ for each free motion interval vs. experimental condition. (**f**–**h**) Average density fields for 12, 18, and 24 moving fast pedestrians respectively. (**i**–**k**) The corresponding fields of the velocity modulus $\langle \left|\overrightarrow{v}\right|\rangle$. The value of $\langle \overrightarrow{v}\rangle$ obtained at each location is indicated by arrows (the black arrow on the top right of figure I corresponds to 1 m/s). (**l**) Distribution of Voronoi areas for three experimental scenarios (“Exp” in the legend), along with the corresponding simulations (“Sim”) when using γ = 1.5 and no turning preference. (**m**) Distribution of Voronoi areas for the experimental case of 18 pedestrians moving fast and the four different scenarios simulated: with (D) and without (ND) damping, with (TP) and without (NTP) turning preference. Also, the Voronoi areas obtained in an elastic gas of spheres are reported for reference.

In recent experiments that we conducted to investigate the effect of social distancing in pedestrian motion^10^, the emergence of CCW rotation came out as a serendipitous discovery when we analysed the velocity fields (Fig. 1i–k). In these tests, participants were freely roaming within a rectangular enclosure, keeping social distancing at different densities and walking speeds (fast or slow)—see (Fig. 1a) and “Methods” for a complete description. We initially thought that the CCW rotation could be triggered by an artefact in the design that broke the symmetry of the system, but this idea was ruled out after a careful inspection of the experimental procedures and setup.

In Fig. 1b and Movie S1, we show the temporal evolution of the crowd instantaneous angular momentum ${\varvec{L}}(t)$ for a given experimental realization $({\varvec{L}}(t)>0$ corresponds to pedestrians rotating CCW around the centre of the arena on average). In the experiment, three “free motion” intervals of about 40 s were interspersed by two intervals of “directed motion”. Free motion (the only scenario analysed here) is found to be correlated with positive values of ${\varvec{L}}(t)$, which also transpires in the distributions of ${\varvec{L}}(t)$ for the different experimental conditions represented in Fig. 1c,d. Averaging ${\varvec{L}}(t)$ over time for 6 different realisations under identical settings confirms the robust and repeatable development of a CCW rotation in the freely moving crowd (Fig. 1e). The increase of the average value $\langle L\rangle$ with the prescribed walking speed is consistent with the linear dependence of ***L*** on speed, while the number of pedestrians (***Ped***)*—*i.e., the density—has a negligible effect, at least within our experimental resolution. Besides, we notice fairly homogeneous walking speeds, with small dips near the corners (Fig. 1i–k), and slightly higher pedestrian densities near the boundaries (Fig. 1f–h). Note that a penchant to walk near walls and boundaries has also been brought to light in assemblies of animals, such as ants^11^.

### Numerical simulations

To shed light on the origins of these observations, we resort to minimal numerical models in which we simulate simple particles self-propelling with a desired speed (without any specific target) and interacting via a long-range repulsive force, to abide by social-distance rules. Short-range (granular) contact forces are also implemented in order to avoid collisions, but hardly ever come into play because of the long-range repulsive forces. A similar scheme is used to model the interactions between particles and walls, for which we also explored the effect of adding a dissipative term, inspired by the observation of higher densities near the arena boundaries similar to those reported for granular media^12^.

To specify the model parameters, we analysed the pedestrian occupation of the space (quantified by their Voronoi areas). The desired velocity and, above all, the long-range repulsive force were found to be essential to reproduce the high homogeneity detected in the experiments. If their magnitudes are properly tuned, the model successfully accounts for the distributions of Voronoi areas for different crowd densities, as shown in Fig. 1l for fast walkers (the agreement is equally good for slow pedestrians). In contrast, the specifics of the pedestrian-wall interaction have a negligible effect on the average occupation of space (Fig. 1m).

**Figure 2:**
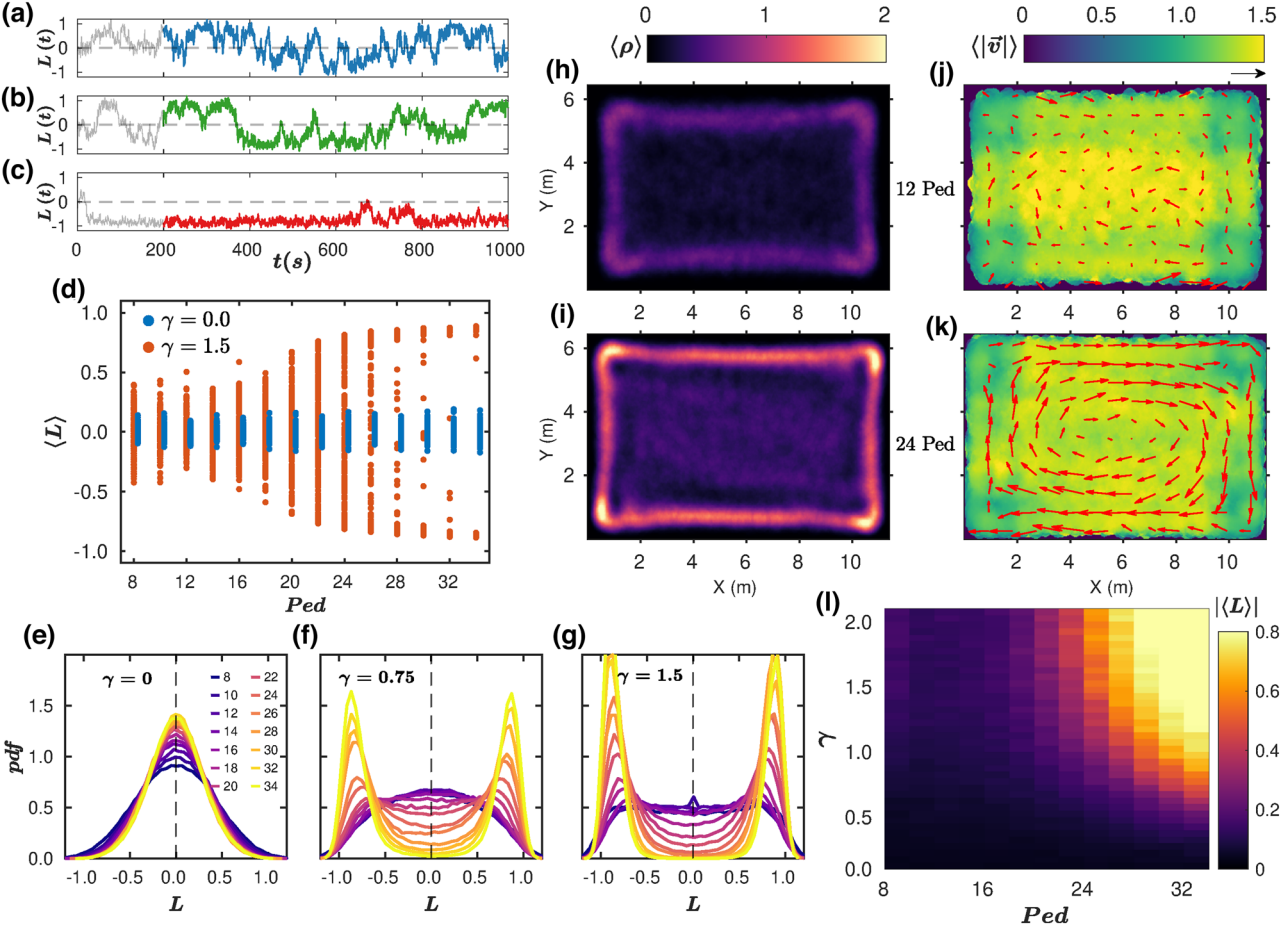
Pedestrian simulations without turning preference. (**a**–**c**) Temporal evolution of the instantaneous angular momentum $L(t)$ for 12 (panel A), 18 (panel B) and 24 (panel C) agents interacting dissipatively with the boundary (γ = 1.5). The time intervals considered in our analysis to avoid the inclusion of a possible initial transient (grey) are indicated with solid colours. (**d**) Bifurcation diagram in which the temporal averaged value of $L(t)$ obtained for each simulation run is represented as a function of the number of pedestrians in the arena (100 data points for each). Different colours correspond to simulations for γ = 1.5 and γ = 0. (**e**–**g**) Distributions of the instantaneous angular momentum $L(t)$ for γ = 0 (panel E), γ = 0.75 (panel F) and γ = 1.5 (panel G), for different number of simulated pedestrians (see legend of panel E). (**h**,**i**) Average density fields obtained for 12 (panel H), and 24 (panel I) pedestrians. (**j**,**k**) The corresponding velocity fields $\langle \left|\overrightarrow{v}\right|\rangle$. Arrows show the value of $\langle \overrightarrow{v}\rangle$ obtained at each location (the arrow on the top right of panel J corresponds to 1 m/s). (**l**) Vortex-like motion phase space. The absolute values of the angular momentum averaged for all frames and the 100 runs for each simulated condition are represented with different colours as a function of the damping parameter and number of pedestrians.

On the other hand, implementing a dissipative interaction (**D**) among pedestrians and walls (by means of a damping coefficient $\gamma >0$) is instrumental in capturing the high densities near the boundaries (Fig. 2h,i). Furthermore, the corresponding velocity fields (Fig. 2j,k) show that, contrary to the undamped (**ND**) situation, vortex-like patterns can then be formed, provided that the crowd is dense enough. In Fig. 2a–c, the temporal evolution of the angular momentum ${\varvec{L}}(t)$ is shown for simulations with $\gamma =1.5$ and three different crowd densities; their mean values, averaged over time and over 100 realizations in identical conditions, are plotted in Fig. 2d as a function of the number of pedestrians in the arena (***Ped***). These figures confirm the transition towards a state of CW or CCW collective vortical circulation as the density increases, provided that the pedestrian–wall interaction is dissipative. Otherwise ($\gamma =0$), values of $\langle L\rangle \sim 0$ reflect the absence of collective rotation. This distinction is mirrored in the different shapes of the distributions of ${\varvec{L}}(t)$ represented in Fig. 2e–g for different crowd densities and damping coefficients, evolving from a single peak centered on ${\varvec{L}}=0$ for $\gamma =0$, to a bimodal distribution for $\gamma =0.75$ if the crowd is dense enough. As the dissipation increases ($\gamma =1.5$) the peaks in the bimodal distribution narrow and the local minimum at ${\varvec{L}}=0$ gets deeper. Therefore, one can conclude that a system of particles with very simple interaction rules is able to trigger vortex-like motion provided that the confining walls are dissipative and the number of agents is sufficiently large to give rise to stable collective motion. This is best seen in the plots of Fig. 2d and in the phase-space diagram of Fig. 2l, where the absolute value of $\langle L\rangle$ is used to gauge the tendency of the system to display vortex-like motion.

### Effect of individual turning preference in the collective behavior

**Figure 3:**
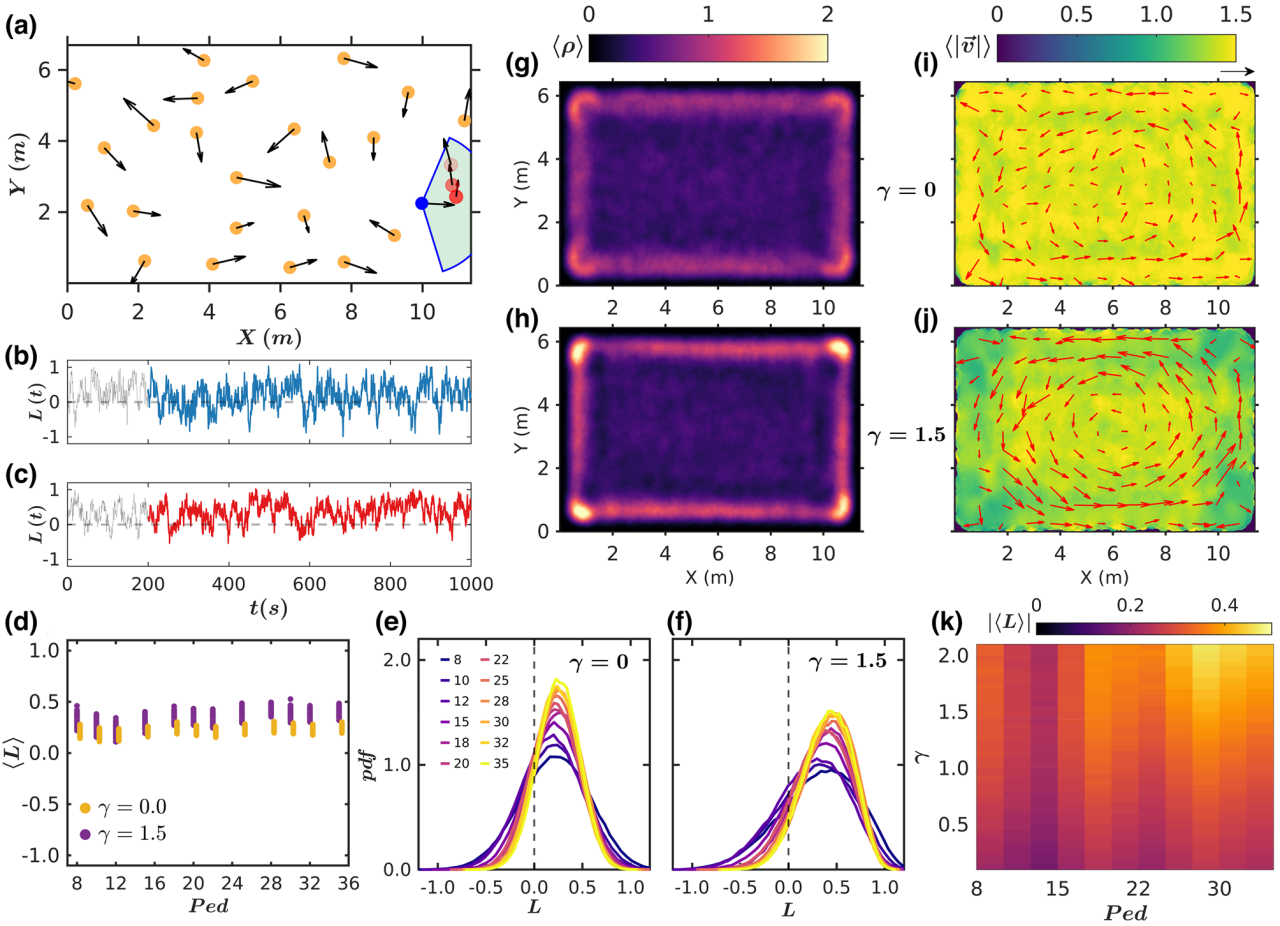
Pedestrian simulations with turning preference. (**a**) Sketch of a single experimental measurement of the turning preference. The trajectory of the analysed pedestrian is shown by circles of different colours (blue for the present position and red, orange and pink for positions after 1, 1.5 and 2 s respectively). The shadowed area corresponds to the region that must be empty to take into account this event (see methods). (**b**,**c**) Temporal evolution of the instantaneous angular momentum $L(t)$ averaged over all pedestrians in the arena for simulations of 12 (panel B), and 24 (panel C) pedestrians with a preferred turning preference and dissipative boundary interaction. The time lapses considered in our analysis to avoid the inclusion of a possible initial transient are shown in solid colours. (**d**) Bifurcation diagram in which the temporal averaged value of $L(t)$ obtained for each simulation run is represented as a function of the number of pedestrians in the arena (100 data points for each simulated condition). All simulations include turning preference and different colours correspond to cases with and without pedestrian-boundary damping, as indicated in the legend. (**e**,**f**) Distributions of the instantaneous angular momentum $L(t)$ when γ = 0 (panel E), and γ = 1.5 (panel F), for different numbers of simulated pedestrians (see legend of panel E). (**g**,**h**) Average density fields obtained for 24 agents when γ = 0 (panel G) and γ = 1.5 (panel H). The corresponding fields of the velocity modulus averaged for each case $\langle \left|\overrightarrow{v}\right|\rangle$ are shown in (**i**,**j**). The value of $\langle \overrightarrow{v}\rangle$ obtained at each location is indicated by arrows (the black arrow on the top right of panel I corresponds to 1 m/s). (**k**) Vortex-like motion phase space. The absolute values of the angular momentum averaged for all frames and the 100 runs for each simulated condition are represented with different colours (see colour bar for reference) as a function of the damping parameter and number of pedestrians.

Still, the foregoing simulations with velocity damping at the wall manifestly fall short of faithfully reproducing some salient experimental features, in particular the breaking of the CW-CCW symmetry. Previous studies performed on chiral particles^13,14^ show that any small tendency to rotate in a particular direction at the level of individual agents would be able to bias the rotation in a certain direction. For our particular case, we claim that this missing ingredient, in complement to (or in replacement for) wall damping, is the propensity of right-handed people to turn left when they come to face a wall, an effect first established in behavioural neuroscience by Mohr et al.^15^ but still debated^16^. In our experiments, by analysing the trajectories of pedestrians who were walking straight to a wall without other individuals in their surroundings (see Fig. 3a and “Methods”), we estimated the preference for right turns to around 60%, not far from the 65% that one would expect in a population with 85% of right-handers (as ours) that would turn left 72% of the times as reported by Mohr et al.^15^. To mimic these turning preferences (TP), a force is introduced parallel to the walls, which promotes a rotation to the left for 60% of the agents and to the right for the remaining 40% upon a head-on incidence on the wall.

The first remarkable effect of adding this force is to inhibit the stable rolls that were previously observed for the densest crowds with damping at the wall. This transpires when comparing the ${\varvec{L}}(t)$ time series in Figs. 2c and 3c, and also the $\langle L\rangle$ vs. ***Ped*** diagrams of Figs. 2d and 3d: switching on the TP substantially reduces the values of $\langle L\rangle$ at the higher densities for $\gamma =1.5$. This can be understood if one regards the agent-dependent turning force as a source of noise, vaguely similar to the addition of a random field to an Ising model, which inhibits the transition to order, as compared to the zero-field counterpart^17^. At the same time, the moderately larger number of agents endowed with left-TP (60% vs 40%) breaks the CW-CCW symmetry, so the likelihood of finding the crowd rotating clockwise ($\langle L\rangle <0$) vanishes. Interestingly, with TP, dissipation at the wall is no longer necessary for the formation of a vortex (Fig. 3d), even though for $\gamma =0$ the values of $\langle L\rangle$ are slightly (but systematically) below those obtained for $\gamma =1.5$. The evolution of these features with $\gamma$ and **Ped** is reported in the diagram of $\left|\langle L\rangle \right|$ shown in Fig. 3k as well as in the distributions of ${\varvec{L}}(t)$ (Fig. 3e,f). The latter are all single peaked, centered at positive values of ${\varvec{L}}(t)$, and very similar for both $\gamma =0$ and $\gamma =1.5$. In comparison with the NTP case, the system becomes less dependent on the damping and the pedestrian number, although in the denser cases the vortex-like motion tends to become stronger with damping [$\gamma =1.5$ (Fig. 3j)] than without it (Fig. 3i), as already reported in Fig. 3d. Moreover, as expected, the concentration of pedestrians near the boundaries is higher in the presence of wall damping (Fig. 3g,h).

## Discussion

All in all, the comparison of the simulation results in different conditions (D vs ND and TP vs NTP) with the experimental findings highlights the need to break the turning symmetry at the wall in order to replicate the experimental results. With this ingredient and a long-range repulsion force among the individuals, the main experimental features are qualitatively reproduced, whether it be the spontaneous development of CCW rotating motion, the single-peaked distributions of angular momenta, the negligible dependence of the angular momenta on the number of pedestrians within the arena, or the enhanced density near the boundaries. Importantly, unlike many previous reports^6–8^, vortices emerged in the absence of ‘flocking’ terms in the interactions that explicitly promote alignment. This new approach complements the one previously presented by Grossman et al.^18^ in which the emergence of coherent rotation was produced as a result of the inelastic collisions between particles.

Of course, it is important to highlight that these results were obtained with a minimal model that undoubtedly fails to capture the full decision-making process in humans. Nevertheless, rather than a limitation, this prompts a generalization of the findings to other situations and possibly other systems, provided that they feature the same minimal ingredients. In particular, Silverberg et al.’s observation that 95% of circle pits at heavy-metal concerts rotate CCW^6^ may be rationalized along the same lines, considering that the circular boundary formed by passive agents around the pit also induces turning preferences. Finally, one could also envision systems composed of particles (synthetically designed^19,20^ or natural) that exhibit some turning preference. For example, some animals are known to display handedness^21^, so it is likely that similar patterns to these reported here would be observed.

Moreover, the findings reported here may also call for additional experimental tests in which the hypothesis of right handers preferring turning right is fully validated and quantified. For example, by controlling the number of left/right handers in each test or -even better- by tuning the number of left/right turners and analyzing the effect of this proportion in the emergence of rotating motion.

## Materials and methods

### Experimental tests

The experiments were performed on 23rd June 2020 in a University building. All methods were carried out in accordance with the guidelines and regulations applying at that time by the regional (Navarra) and national (Spain) Governments. In particular, all participants had to wear a mask during the 3 h that the experiments lasted and use hydroalcoholic gel. Finally, all experimental protocols were previously approved by the University of Navarra ethics committee and informed consent was obtained from all participants.

The volunteers (28 men and 10 women) were mainly right-handed (85%) and aged between 19 and 59 years. From these people, different subgroups were sequentially asked to walk within a rectangular arena (11.4 m wide by 6.7 m long) while keeping a prescribed distance of 2 m at a given speed (either fast, F, or slow, S, depending on the test). The effect of pedestrian density was investigated by implementing tests with subgroups of 12, 18 and 24 participants walking within the enclosure. For the purpose of checking the reproducibility of the experiments, each condition was repeated twice with different people. In this way, a total of 12 runs were performed for the 6 different experimental scenarios. In the original tests, the effect of reducing the prescribed safety distance to 1.5 m was also explored, but the outcomes obtained in these experiments are not relevant for this work as they are comparable to those for a distance of 2 m.

For each run, the procedure was the following: first, pedestrians entered the arena and took their place at one of the spots marked on the floor at a distance of 2 m from the rest. These marks also served as a reference for participants to estimate the social distance they had to keep away from others. Then, the volunteers were asked to roam within the enclosure keeping the prescribed safety distance and avoiding stopping as much as possible. For the case of slow walking, they were asked to roam peacefully, as if they were window-shopping in the street with no rush at all. In the case of fast walking, they were just requested to walk fast, without further explanation. After about 40 s, people were asked to walk towards one of the four walls, before resuming the random motion. This procedure was repeated again after a further 40 s and finally people stopped walking at about 120 s since the start of the run. In this way, three phases of “free motion” were interspersed with two periods of movement toward the walls, in an attempt to break up the possible appearance of organized collective movements. As in previous works^10,22^, only the stages of “free motion” are considered in our study (see green line in Fig. 1b).

All experiments were recorded with a 4 K resolution camera at a frame-rate of 25 fps. A home-made image analysis program was used to track the positions and velocities of all pedestrians on the videos. The latter were calculated over a sliding window of 0.76 s to ensure that pedestrians had moved a reasonable distance, hence reducing spurious noise. The same strategy was followed to compute the Voronoi area corresponding to each pedestrian, as explained in Ref.^19^. From the positions and velocities of pedestrians we computed the mean density $\rho (\overrightarrow{r},t)$ and velocity fields $\overrightarrow{v}(\overrightarrow{r},t)$ using a 2D Heaviside coarse graining function $\Omega (\overrightarrow{r}-{r}_{i}(t)) = H(\omega -|\overrightarrow{r}-{\overrightarrow{r}}_{i}(t)|)/\pi {\omega }^{2}$, with $\omega$ = 0.25 m. In this way, the contribution of each pedestrian to these fields does not take place at a single point, but spreads over a larger region about the physical size of one person. Finally, mean fields have been obtained through the time average of the fields for all frames in each configuration, i.e*.*, $\rho (\overrightarrow{r})=<\rho (\overrightarrow{r},t)>$ and $\overrightarrow{V}(\overrightarrow{r})=<|\overrightarrow{v}(\overrightarrow{r},t)|>$.

### Angular momentum

To characterize the collective rotation, we have defined the normalized angular momentum $\overrightarrow{L}(t)$ of the system as:

1 $$L(t) = \frac{1}{N}{\sum }_{i=1}^{N}\frac{{\overrightarrow{r}}_{i}\left(t\right)\times {\overrightarrow{v}}_{i }\left(t\right)}{\left|{\overrightarrow{r}}_{i}\left(t\right)\right|},$$

where $N$ is the total number of people within the arena, ${\overrightarrow{v}}_{i}$ the velocity of pedestrian $i$, and ${\overrightarrow{r}}_{i}$ its position (with the origin at the centre of the arena). The only non-zero component of $\overrightarrow{L}(t)$ is normal to the plane of motion, and we refer to it as ${\varvec{L}}(t)$. Note that the angular momentum is normalized both by the number of people and by their distance to the centre of the enclosure. Thus, each pedestrian contributes equally to the angular momentum, irrespective of their position. Importantly, we have checked that the results obtained with Eq. (1) are qualitatively the same if we do not divide by $\left|\overrightarrow{{r}_{i}}(t)\right|$.

### Turning preference

The turning preference was experimentally estimated by means of a careful analysis of the pedestrian trajectories. From this, we decided to consider only turns taking place when pedestrians faced a wall more or less perpendicularly, and in the absence of neighbours or other walls that might affect the turning direction. Thus, the turns had to fulfill four conditions to be taken into account in the analysis:

1. The turn must take place at two meters or less from the facing wall.
2. Turns performed at a distance closer than 2 m from any of the four corners were not considered. This is because the movements near the corners are limited (or highly conditioned) by the spatial confinement.
3. To ensure that the presence of other people did not influence the turn, the pedestrian field of view had to be empty of other pedestrians. We define it as a circular sector of 2 m radius and ± 70° aperture^23^, with the origin of angles given by the pedestrian velocity (see the green region in Fig. 3a).
4. Finally, the angular difference α between pedestrians' velocity and the vector normal to the wall (Fig. S1b) should be smaller than ± 10° (meaning that the agents walk almost perpendicularly to the wall). By doing this, we avoided considering turns that were somehow conditioned on the pedestrian's direction of motion.

For all the pedestrians who met the above conditions, the evolution of the trajectory was analysed over a window of two seconds and the turn was labelled as either CCW or CW (red dots in Fig. 3a mark the different positions analysed). In this way, a total of 75 turns were evaluated from which 43 (57.3%) were CCW and 32 (42.7%) CW.

### Numerical simulations

#### The model

The used numerical framework is a minimal model of self-propelled particles without preferential direction of motion. Thus, the self-propulsion mechanism is driven by the force:

2 $${\overrightarrow{F}}_{i}^{Propulsion }= \mu \left({v}_{d}-{v}_{i}\right){\widehat{v}}_{i},$$

where ${v}_{i}$ and ${v}_{d}$ are the current and the desired speeds respectively, the unity vector ${\widehat{v}}_{i}$ is the agent moving direction, and $\mu$ is a constant that sets the magnitude of the self-propelling force. Note that the self-propulsion force always acts in the direction of motion. Consequently, the direction can only be changed by possible interactions with other agents or with the boundaries, as explained below.

The pedestrian interaction is modelled using a long-range and a short-range repulsion term. The former attempts to qualitatively reproduce the phenomenon of social distancing while the latter is a Hertzian (granular) repulsion force^24^ that prevents overlapping. The resulting force reads:

3 $${\overrightarrow{F}}_{i}^{Rep-Ped }= \left\{\begin{array}{l}{A}_{P} \cdot  exp\left(-\frac{({r}_{ij}-2{r}_{0})}{{B}_{p}}\right)\widehat{n} \quad { r}_{ij} >2{r}_{0} \\ \varepsilon {\left(1-\frac{{r}_{ij}}{2{r}_{0}}\right)}^{3/2}\widehat{n} \quad otherwise.\end{array}\right.$$

The long-range force has an exponential form that decays with the distance between the interacting agents. This is computed as the distance between the agents’ positions ${r}_{ij}$ minus twice the agents radius ${r}_{0}$. As depicted in Fig. S1a, ${r}_{ij}$ is measured in the normal direction $\widehat{n}$, a unit vector defined as the line connecting the centres between particles $i$ and $j$. The force amplitude ${A}_{P}$ and the characteristic decay length ${B}_{p}$ are chosen to reproduce the experimental outcomes of the local space occupation by each pedestrian, as explained below. The short-range term is a Hertzian repulsion force of strength $\varepsilon$, also pointing in the direction of $\widehat{n}$. The role of the latter is barely relevant in our simulated scenario given that the long-range term dominates the interactions.

The particles are also affected by the walls experiencing a repulsion force that follows the same scheme as the inter-particle one. Again, we combine long and short-range terms that depend on the relative position between the agent and the closest point to the wall. Additionally, inspired by the observation of higher densities near the walls, a dissipative term in the particle–wall interaction has been introduced, thus reading:

4 $${\overrightarrow{F}}_{i}^{Rep-Wall }= \left\{\begin{array}{l}\left[{A}_{w}^{1} \cdot  exp\left(-\frac{({r}_{iw}-{r}_{0})}{{B}_{w}}\right)-\gamma {v}_{i}^{n}\right] \cdot  \widehat{n} \quad {r}_{iw} >{r}_{0}\\ \varepsilon {\left(1-\frac{{r}_{iw}}{{r}_{0}}\right)}^{3/2}\widehat{n} \quad otherwise,\end{array}\right.$$

where ${A}_{w}^{1}$ and ${B}_{w}$ are the amplitude and the characteristic decay length of the exponential force respectively, ${r}_{iw}$ is the distance between the particle and the nearest point of the wall, $\gamma$ is the strength of the damping term, and ${v}_{i}^{n}$ the component of the velocity vector projected in the $\widehat{n}$ direction (see Fig. S1b). The short-range repulsive term is again a Hertzian force with the same strength as in Eq. (2).

Finally, in order to recreate the pedestrian turning preference, a new force acting along the wall’s tangential direction $\widehat{t}$ has been added in some simulations:

5 $${\overrightarrow{F}}_{i}^{Turning }= \left\{\begin{array}{l}{A}_{w}^{2} \cdot  exp\left(-\frac{({r}_{iw}-{r}_{0})}{{B}_{w}}\right)cos(\alpha ) \cdot  {\widehat{t}}_{+/-} \quad \left|\alpha \right|<90^\circ \\ 0 \quad otherwise.\end{array}\right.$$

We decided to keep the same exponential form as in Eq. (3) (with different amplitude ${A}_{w}^{2}$), but we multiplied it by two terms. The first one is $cos(\alpha )$, being $\alpha$ the angle between ${\widehat{v}}_{i}$ and $\widehat{n}$, and it accounts for the fact that the turning force should be maximum when pedestrians face the wall perpendicularly and vanish when walking parallel to it. Also, when the agents move away from the wall (|$\alpha |$> 90°) the turning force is zero. The second term ${\widehat{t}}_{+/-}$ determines the rotation direction which is predefined for each agent (and unchanged over each simulation run): for ${\widehat{t}}_{-}$ the agent prefers turning CW, while for ${\widehat{t}}_{+}$ the agent prefers turning CCW (see Fig. S1b).

#### Parameters choice

Parameters were selected to mimic, in a qualitative way, some of the most salient experimental features. First, the radius for all particles was set to ${r}_{0}=0.25 m$. Then, for the self-propulsion term, we looked at the probability density functions (pdfs) of the actual speeds^10^ and, with the appropriate units of SI, we set $\mu =4$ and ${v}_{d}=1.5.$ Regarding the parameters governing the repulsion between pedestrians, we focus on replicating the rather homogeneous occupation of space observed in the experiments. Taking the pdfs of the Voronoi areas as a reference, we observed that the combination of parameters ${A}_{P}\in (\mathrm{10,15})$ and ${B}_{p}\in (\mathrm{0.5,1})$ offered a nice agreement for the three global densities explored experimentally (Fig. 1l). Thus, we chose the values ${A}_{P}=13$ and ${B}_{p}=0.85$. As for the short-range force, it has little effect on the global dynamics. A value $\varepsilon =200$ was set in order to avoid large overlaps (> 5%).

Concerning the interaction with the walls, the repulsion term has the same short-range force as for pedestrians, while for the long-range force we set the characteristic length to ${B}_{w}=0.4$. In this way, we reproduce the experimental observation that closer approaches to the walls are more frequent than among pedestrians. Accordingly, and in an attempt to prevent unwanted collisions between agents and walls, the amplitude of the force was increased to ${A}_{w}^{1}= 15$.

Lastly, the choice of the amplitude for the turning mechanism was made by drawing inspiration from the experimental procedure implemented to compute the turning preference. Recall that we considered all pedestrians facing the walls at an angle smaller than ± 10° and without nearby persons, obtaining 57.3% CCW turns and 42.7% CW. Then, provided that our intention is keeping the same value of exponential decaying length ${B}_{w}$ used for the pedestrian-wall repulsion term, we calculated the value of ${A}_{w}^{2}$ needed to induce a CCW rotation if an agent with ${\widehat{t}}_{+}$ faced the wall with $\alpha =-10^\circ$ (or, to induce a CW rotation if an agent with ${\widehat{t}}_{-}$ faced the wall with $\alpha =+10^\circ$, which amounts to the same). By running simulations with just a single agent (no interaction with other pedestrians) and one wall, we obtain that a good choice for this value is ${A}_{w}^{2}$ = 9. Then, in each simulated run, we set a proportion of agents with ${\widehat{t}}_{+}$ and ${\widehat{t}}_{-}$ as close as possible to 60% and 40%. In this way, the turning preference statistics are similar to the experimental ones.

#### Simulation protocol

An arena identical to the experimental one was simulated: a rectangular enclosure of 11.4 m wide by 6.7 m long. Different scenarios were simulated varying the global density of the system (from ***Ped*** = 8 to ***Ped*** = 34 pedestrians, one at a time) and the damping coefficient (from $\gamma$=0 to $\gamma$=2, every 0.05). For each pair of values (Ped_i_,${\gamma }_{i}$) a total of 100 repetitions were performed varying the initial conditions (pedestrian positions and orientations); hence leading to ${10}^{5}$ total runs. In each run, 1000 s of motion were simulated. Then, we calculated the temporal evolution of the angular momentum following Eq. (1). Also, the overall rotation was characterized by calculating the temporal average $\langle L\rangle$ for each run but excluding the first 200 s of each run to prevent the inclusion of a possible initial transient in the dynamics (see Figs. 2a–c and 3b,c).

Finally, let us note that the numerical integration of $m{\ddot{\overrightarrow{r}}}_{i} ={\overrightarrow{F}}_{i}^{Propulsion }+{\overrightarrow{F}}_{i}^{Rep-Ped }+{\overrightarrow{F}}_{i}^{Rep-Wall }+{\overrightarrow{F}}_{i}^{Turning }$ was performed using a forward Euler method^6,25^ with a time step dt = 0.01 s. Lower values of dt were also used to test the convergence of the implemented framework.

## Supplementary Information

Supplementary Figure 1.

Supplementary Video 1.

## Data Availability

Source data and numerical code are available at: http://ped.fz-juelich.de/extda/echeverria-huarte2020. https://github.com/pedestrianman/VortexPedestrian.
