# Supplementary material for: Spontaneous emergence of counterclockwise vortex motion in assemblies of pedestrians roaming within an enclosure
Source: Sci Rep. 2022 Feb 16;12:2647. doi: 10.1038/s41598-022-06493-0 (PMC8850453; doi:10.1038/s41598-022-06493-0)
Supplement: Supplementary file 1 — Supplementary Figure 1. [file 41598_2022_6493_MOESM1_ESM.pdf]

Supplementary Information for:

Spontaneous emergence of counterclockwise vortex motion in assemblies of pedestrians roaming within an enclosure.

I. Echeverría-Huarte[1], A. Nicolas[2], R.C. Hidalgo[1], A. Garcimartín[1], I. Zuriguel[1*]

[1]Departamento de Física y Matemática Aplicada, Facultad de Ciencias, Universidad de Navarra, 31080 Pamplona, Spain.
[2]Institut Lumière Matière, CNRS & Université Claude Bernard Lyon 1& Université de Lyon, F-69622, Villeurbanne, France.

Abstract

We provide one additional figure and a video as Supplemental Material:

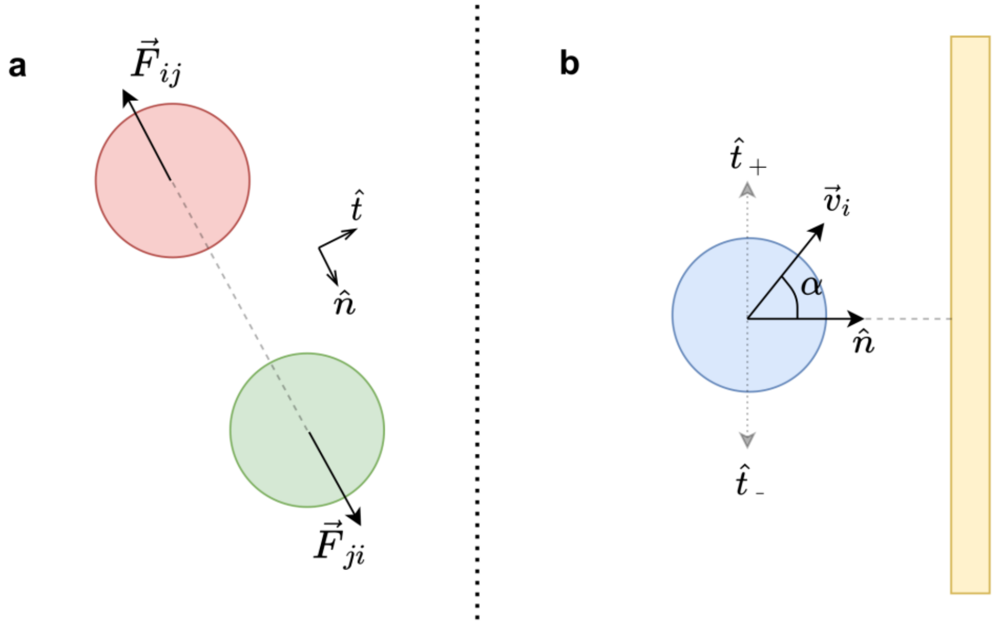

Supplementary Figure 1. Interaction sketch and vectors definition. (a) Contact interaction between two particles. **(b)** Contact interaction between the particle and the wall.

Supplementary Video 1. Crowd Instantaneous angular momentum. Temporal evolution of the instantaneous angular momentum **L**(t) average over 24 pedestrian in a fast walking speed experiment. The tails show the individual measure $L_i(t)$ for each agent over the last 1.5 seconds. They have been coloured according to the colorbar on the right.
